# Transcriptome Profiles Reveal the Crucial Roles of Auxin and Cytokinin in the “Shoot Branching” of *Cremastra appendiculata*

**DOI:** 10.3390/ijms19113354

**Published:** 2018-10-26

**Authors:** Xiang Lv, Mingsheng Zhang, Xiaolan Li, Ruihua Ye, Xiaohong Wang

**Affiliations:** School of Life Sciences/State Engineering Technology Institute for Karst Desertification Control, Key Laboratory of Plant Resources Conservation and Germplasm Innovation in Mountainous Region (Ministry of Education), Guizhou University, Guiyang 550025, China; 05lvxiang@163.com (X.L.); lixiaolanl@163.com (X.L.); m18275348926@163.com (R.Y.); swuwxhong@163.com (X.W.)

**Keywords:** *Cremastra appendiculata*, shoot branching, transcriptome, phytohormone signaling, transcription factors

## Abstract

*Cremastra appendiculata* has become endangered due to reproductive difficulties. Specifically, vegetative reproduction is almost its only way to reproduce, and, under natural conditions, it cannot grow branches, resulting in an extremely low reproductive coefficient (reproductive percentage). Here, we performed RNA-Seq and a differentially expressed gene (DEG) analysis of the three stages of lateral bud development in *C. appendiculata* after decapitation—dormancy (D2), transition (TD2), and emergence (TG2)—and the annual axillary bud natural break (G1) to gain insight into the molecular regulatory network of shoot branching in this plant. Additionally, we applied the auxin transport inhibitors *N*-1-naphthylphthalamic acid (NPA) and 2,3,5-triiodibenzoic acid (TIBA) to a treated pseudobulb string of *C. appendiculata* to verify the conclusions obtained by the transcriptome. RNA-Seq provided a wealth of valuable information. Successive pairwise comparative transcriptome analyses revealed 5988 genes as DEGs. GO (Gene Ontology) and KEGG (Kyoto encyclopedia of genes and genomes) analyses of DEGs showed significant enrichments in phytohormone biosynthesis and metabolism, regulation of hormone levels, and a hormone-mediated signaling pathway. qRT-PCR validation showed a highly significant correlation (*p* < 0.01) with the RNA-Seq generated data. High-performance liquid chromatography (HPLC) and qRT-PCR results showed that, after decapitation, the NPA- and TIBA-induced lateral buds germinated due to rapidly decreasing auxin levels, caused by upregulation of the dioxygenase for auxin oxidation gene (*DAO*). Decreased auxin levels promoted the expression of isopentenyl transferase (*IPT*) and cytochrome P450 monooxygenase, family 735, subfamily A (*CYP735A*) genes and inhibited two carotenoid cleavage dioxygenases (*CCD7* and *CCD8*). Zeatin levels significantly increased after the treatments. The increased cytokinin levels promoted the expression of *WUSCHEL* (*WUS*) and inhibited expression of *BRANCHED1* (*BRC1*) in the cytokinin signal transduction pathway and initiated lateral bud outgrowth. Our data suggest that our theories concerning the regulation of shoot branching and apical dominance is really similar to those observed in annual plants. Auxin inhibits bud outgrowth and tends to inhibit cytokinin levels. The pseudobulb in the plant behaves in a similar manner to that of a shoot above the ground.

## 1. Introduction

*Cremastra appendiculata* is a rare, medicinal perennial plant. A variety of pharmacologically active compounds, with properties such as anti-angiogenic activity [1], selective blockade activity of muscarinic M3 receptors [2], and antitumor activity [3,4,5,6], can be isolated from its pseudobulb. Therefore, *C. appendiculata* has attracted the attention of scholars. However, scarcity of resources is a bottleneck for the development and utilization of *C. appendiculata*. The rate of fruit set is only 1.3–2.0% in natural conditions [7]. However, its fruit rate can be increased to over 95% with artificially assisted pollination [7]. Its seeds rarely germinate due to their immature embryos and lack of endosperms, a characteristic that has resulted from the loss of many genes [8]. The cause of immature embryo development is still unclear at present. Yagame et al. [9] used a fungal (SI1-1 or KM1-1) co-culture technique to improve the seed germination rate. As a result, more than 30% of the seedlings grew vigorously and developed a rhizome, but, unfortunately, they did not develop into pseudobulbs. Thus, vegetative reproduction is still the main reproductive route for *C. appendiculata* at present.

**Figure 1:**
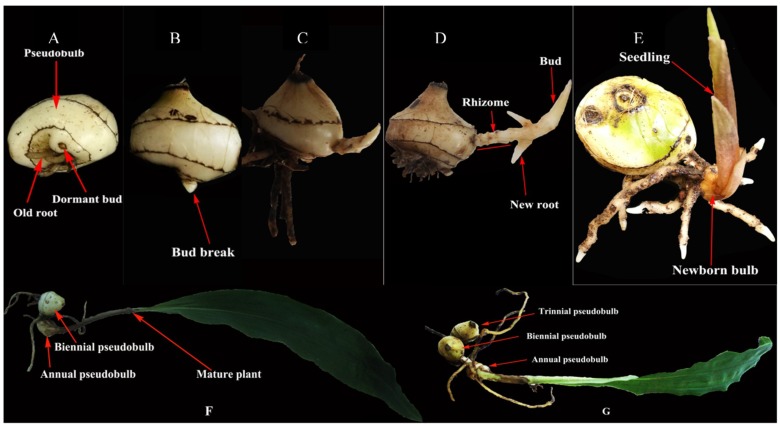
Pseudobulb string formation process: the pictures in (**A**–**F**) illustrate the development and growth processes of a newborn bulb. Annually, *C. appendiculata* forms into a biennial plant (**F**) through this development and growth process. Once per year, the biennial plant grows into a triennial plant through recycling (**G**). After repeated growth cycles, this plant forms a pseudobulb string.

*C. appendiculata*, a typical perennial plant, naturally has a large bud bank, but its latest pseudobulb can only produce one bud, producing one new pseudobulb each year (Figure 1A–F). In fact, the other buds (including the lateral buds) also have the potential for bud elongation and growth [10], and *C. appendiculata* forms a pseudobulb string year after year (Figure 1). In other words, *C. appendiculata* cannot achieve shoot branching, that is the burst of lateral buds, on pseudobulbs string under natural conditions. This results in a very low reproductive coefficient, which in turn limits the development and utilization of *C. appendiculata*. Thus, it is very important to reveal the molecular mechanism that inhibits lateral bud break. In recent years, we have constructed effective techniques to relieve lateral bud inhibition and increase the reproductive coefficient in this plant, and we previously reported that shoot branching of *C. appendiculata* is related to phytohormones (auxin and cytokinin) [10]. However, its molecular regulation mechanisms are still not well understood. 

In this plant, only a small proportion of the buds (from the annual pseudobulb) yield branches under natural conditions. Both the timing and extent of bud activation are tightly controlled to produce specific branching architectures. Shoot branching is a highly plastic developmental trait that is controlled by complex interactions between genetic, hormonal, nutrient, and environmental factors [11,12,13,14,15,16]. The crucial roles of phytohormones in shoot branching are becoming more evident [14,17,18]. It is well known that auxin and cytokinin (CTK) play antagonistic roles in regulating axillary bud outgrowth [14,19]. The primary shoot apex can inhibit the activation of lateral buds through a process known as apical dominance. Auxin was the first hormone reported to be associated with apical dominance [17]. It directly inhibits the biosynthesis of cytokinins through an *AXR1*-dependent auxin signaling pathway [20], and, thus, suppresses axillary bud outgrowth [19]. Experiments have shown that auxin promotes the expression of strigolactone biosynthesis genes [21,22,23,24]. On the contrary, strigolactone can regulate shoot branching via the repression of auxin canalization [25,26,27]. The interactions among these phytohormones regulate axillary bud outgrowth, but their mechanism of interaction in *C. appendiculata* and the related interconnected molecular process is unclear.

Transcription factors (TFs) play key roles in controlling lateral bud growth. *BRANCHED1* (*BRC1*) has been reported as an integrator of branching signals that regulates shoot branching [15,28,29]. *MYELOBLASTOSIS ONCOPROTEIN 13* (*MYB13*) can initiate axillary bud outgrowth in the tomato [30] and modify the architecture of *Arabidopsis* inflorescence [31]. *WRKY TRANSCRIPTION FACTOR 71* (*WRKY71*) regulates auxin homeostasis, which controls shoot branching in *Arabidopsis* [32,33]. However, it is unclear as to whether a consistent regulatory mechanism exists in the shoot branching of *C. appendiculata.*

The transcriptomic approach has been used to analyze reproduction [34], plant growth and development [35,36,37,38], secondary metabolites [39,40], and so on. The transcriptome can not only obtain a large amount of genetic information but can also reveal molecular mechanisms through a differentially expressed gene (DEG) analysis. One of the key issues in transcriptome analysis is ensuring scientific and suitable sampling. For this reason, we previously performed morphological and structural anatomical analyses on the development processes of lateral buds after decapitation. It was identified that the emergence processes of the lateral buds can be divided into three stages, the dormancy stage (D2), the transition stage (TD2), and the lateral bud break stage (TG2), where the number 2 indicates that the sample is from biennial pseudobulbs [10].

To explore the molecular regulatory network of shoot branching, we harvested biennial pseudobulb lateral buds from the three stages (D2, TD2, and TG2) during the lateral bud breaking process and collected the annual axillary buds of the natural breaking stage (G1, where the number 1 indicates that the sample is from annual pseudobulbs) to perform an RNA-Seq analysis. To further validate the results of the transcriptome analysis, the auxin transport inhibitors *N*-1-naphthylphthalamic acid (NPA) and 2,3,5-triiodibenzoic acid (TIBA) were applied to a treated pseudobulb string to analyze the lateral bud development phenotype and changes in hormone levels (indole-3-acetic acid (IAA) and zeatin (ZT)) in lateral buds, and to investigate the expression characteristics of candidate genes. The present investigation provided valuable transcriptome data related to the regulation of the lateral bud break in *C. appendiculata*. Thus, this study lays the foundation for the molecular breeding of *C. appendiculata* and for studying the apical dominance of the underground stem.

## 2. Results

### 2.1. Decapitation and Auxin Transport Inhibitors Affect Lateral Buds Break

**Figure 2:**
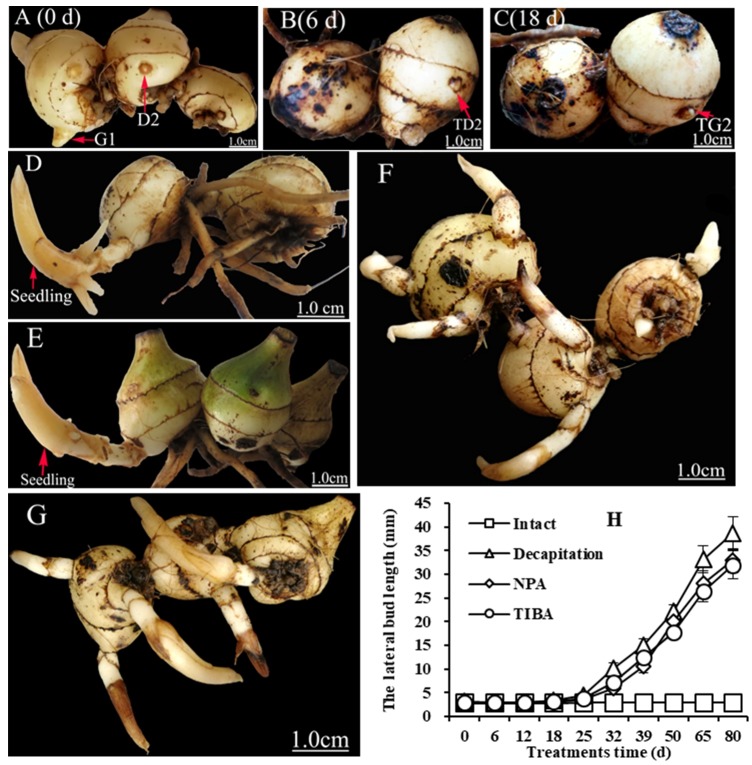
The decapitation, NPA, and TIBA treatments promoted lateral bud outgrowth: (**A**–**C**) morphological pictures of the lateral buds at three representative time points (0, 6, and 18 days post-decapitation); (**D**–**G**) morphological pictures of decapitated and intact plants, and those treated with NPA and TIBA after for 80 days; and (**H**) the statistical chart of bud length. Values are means ± SDs, *n* = 3. Error bars indicate standard deviations obtained from three biological replicates.

In *C. appendiculata*, the annual pseudobulb strongly inhibits lateral bud outgrowth. To induce lateral bud breaking, auxin transport inhibitors (naphthylphthalamic acid (NPA) and 2,3,5-triiodibenzoic acid (TIBA)) and decapitation were used to treat a pseudobulb string. Morphologically, the lateral buds showed no significant changes six days post-treatment (Figure 2A,B,H), a period called the transition stage. At 18 days post-treatment, the lateral bud breaking stage began. The morphological feature of breaking was the emergence of white shoot apices from the lateral buds (Figure 2C). Subsequently, 80 days post-treatment, the lateral buds grew into seedlings (Figure 2D,F,G). These results show that NPA and TIBA have the same effect as decapitation. The lateral buds of intact plants could not break and grow into seedlings (Figure 2E,H), indicating that auxin plays a key role in regulating the lateral bud break in the plant.

### 2.2. Content Changes of Hormones in the Lateral Buds during the Bud Elongation Process

**Figure 3:**
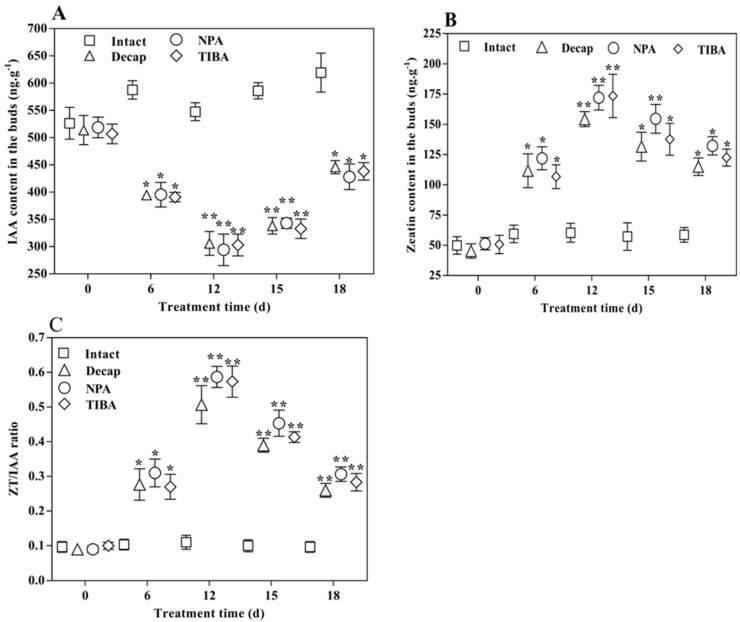
The phytohormone content of *C. appendiculata* lateral buds was tested during the bud elongation process by HPLC: (**A**) IAA; (**B**) zeatin; and (**C**) zeatin/IAA ratio. Values are means ± SD, *n* = 3. Error bars indicate the standard deviations obtained from three biological replicates. * and ** indicate significant differences based on one-way ANOVA tests at *p* < 0.05 and *p* < 0.01, respectively, compared with the intact group.

To confirm whether lateral bud breaking is related to the disruption of the auxin–cytokinin balance, the levels of hormones were analyzed at five time points by HPLC (Figure 3). The IAA (Indole-3-acetic acid) content was the highest at the D2 stage, decreased significantly six days post-treatment (*p* < 0.05), and then increased gradually 15 days post-treatment (Figure 3A). As expected, opposite trends were observed for IAA and zeatin (ZT) (Figure 3B). The ZT/IAA ratio varied with changes in the content of each (Figure 3C). These results indicate that changes in the auxin content cause the variation in cytokinin levels, and disruption of the auxin–cytokinin balance is necessary for lateral bud break.

### 2.3. Sequence Analysis, Read Assembly, and Annotation

To get an overview of the regulatory networks of shoot branching, cDNA libraries were generated from RNA isolated from 12 samples (G1_1, G1_2, G1_3, D2_1, D2_2, D2_3, TD2_1, TD2_2, TD2_3, TG2_1, TG2_2, and TG2_3) and subjected to paired-end sequencing on the Illumina platform. The raw read sequences have been logged in NCBI’s sequence read archive (SRA) (BioProject ID PRJNA474994). A de novo assembly strategy was executed as *C. appendiculata* lacks a reference genome sequence. In total, 618,793,678 raw reads were generated from 12 samples. After cleaning and quality checks, 597,053,172 high-quality clean reads were assembled into 239,732 genes with a mean length of 921 bp and an N50 length of 1282 bp (Table 1).

The BSCO (Benchmarking Universal Single-Copy Orthologs) assessment showed many single-copy genes (*S* = 80.3%, *F* = 4.0%, and *C* = 86.6%; Table S1), indicating that the assembly integrity of these genes was good. The PCA (Principal components analysis) analysis showed that the four samples were not heterozygous (Figure S1). The biological replicate samples were also gathered together (Figure S2). The Q20 percentage (sequencing error rate <1%) for the clean reads was over 96%, and the GC content was above 45% for the set of libraries (Table 1). The length distribution of the assembled unigenes showed that there were 137,279 genes (57.26%) in the range of 301–1000 bp; 49,961 genes (20.84%) in the 1100–2000 bp range; and 21,878 genes (9.13%) with a length of >2000 bp (Figure S3). More details are shown in Table 1 and Figure S3.

**Figure 4:**
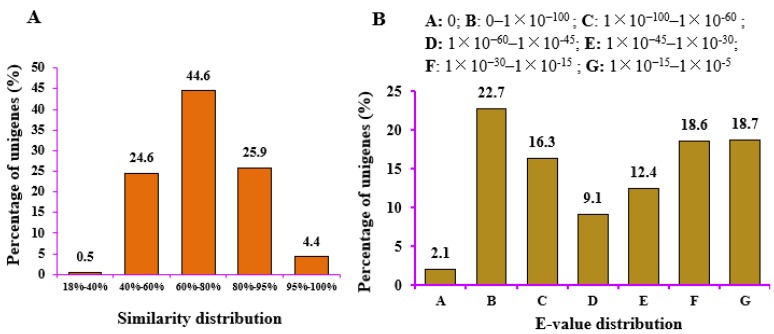
Characteristics of the similarity search of unigenes against the NR database: (**A**) similarity distribution of the top BLAST hit for each gene; and (**B**) E-value distribution of BLAST hits for each unigene with a cutoff E-value.

A total of 129,293 genes (53.93%) matched at least one database (Table S2). Statistical analysis of the annotated unigenes in the NR database revealed strong homology, with an E-value smaller than 1 × 10^−60^ for 45,447 genes (41.1%), while the remaining 58.9% of the genes had high homology (1 × 10^−60^ < E-value < 1 × 10^−5^) (Figure 4A). A similarity analysis showed that genes with extremely high similarity (similarity > 80%) accounted for 30.3% (33,505 genes), highly similar genes (similarity between 60% and 80%) accounted for 44.6% (49,318 genes), and similar genes (similarity below 60%) accounted for 25% (Figure 4B). These results indicate that these genes had high-confidence hits. 

The overall quality of the RNA-Seq was assessed with a Pearson correlation analysis of gene expression across samples. The results showed that the correlation coefficients of the sample between biological replicates were greater than those of the samples of non-biological replicates (Figure S4).

### 2.4. Successive Pairwise Comparisons of DEG Profiles

To understand the relationship between the lateral bud development phenotype and DEGs, pairwise comparisons were performed between the four stages (G1, D2, TD2, and TG2). In total, 5988 DEGs were obtained via pairwise comparisons (Table S3). The results showed that TD2 vs. D2 and G1 vs. TG2 had few DEGs: 67 and 110, respectively. As expected, G1 vs. TD2 and TG2 vs. TD2 had more DEGs: 3843 and 3031, respectively. Additionally, of the TG2 vs. TD2 and TD2 vs. D2 comparisons, TG2 vs. TD2 had the greater number of DEGs. This indicates that TG2 and G1 are critical periods of bud development. For more detailed information, please refer to Table S3 and Figure S5. A Venn diagram was constructed to exhibit the relationships between DEGs in the three stages (dormancy, transition, and bud break) (Figure S6). The results show that 97.5% DEGs (3015 genes) had specific differential expression and 0.52% DEGs (16 genes) had common expression. This further illustrates that the transcriptome changes dramatically from the TD2 to TG2 stages.

### 2.5. GO (Gene Ontology) and KEGG (Kyoto Encyclopedia of Genes and Genomes) Enrichment Analyses of All DEGs

**Figure 5:**
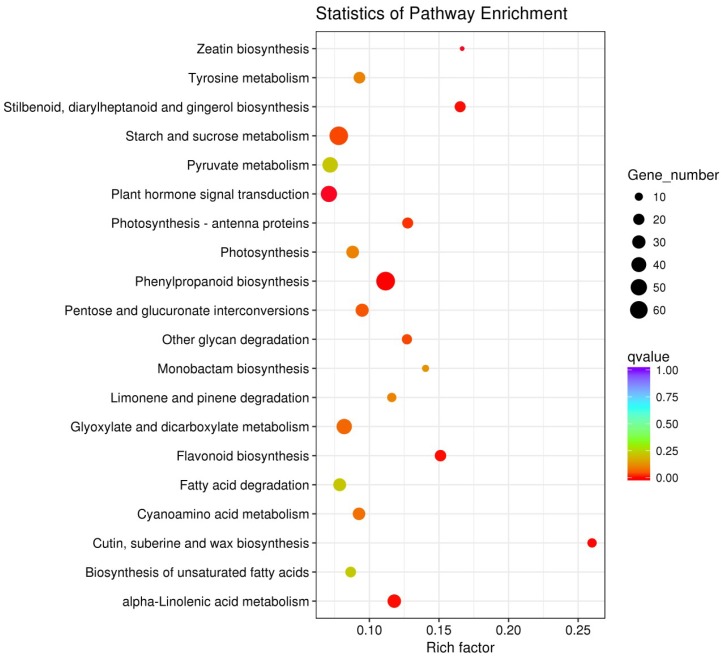
KEGG enrichments of the annotated differentially expressed genes (DEGs). The left *Y*-axis indicates the KEGG pathway. The *X*-axis indicates the rich factor (the number of genes enriched in this pathway compared to the number of genes on this pathway.). High *q* values are shown in blue, and low *q* values are shown in red.

To predict the relationships between differentially expressed genes and biological processes and their functions, DEGs were annotated by GO and KEGG analyses to examine the putative functional differences between different successive developmental stages. Significantly enriched GO terms (corrected *p*-value < 0.05) were involved in 140 GO terms. Among these, nine GO terms were related to hormone metabolism and signal transduction (Table 2). The mapping of all DEGs to terms in the KEGG database resulted in significantly enriched terms (corrected *p*-value < 0.05) in 11 pathways (Figure 5). Of these pathways, two (zeatin biosynthesis and plant hormone signal transduction) were associated with hormone metabolism and signal transduction. These results surmise that the phytohormones are involved in the lateral bud break in the plant.

### 2.6. Cluster Analysis of Hormone- and Transcription Factor-Regulated DEGs during the Bud Elongation Process

**Figure 6:**
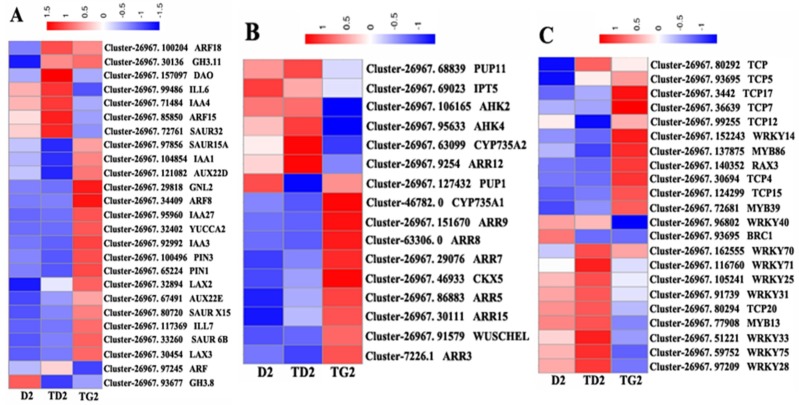
Hierarchical cluster analysis of auxin- (**A**), cytokinin- (**B**), and transcription factor-related (**C**) DEGs during lateral bud elongation in *C. appendiculata*. Red indicates high relative gene expression and green indicates low relative gene expression.

As shown in Figure 6, these genes were expressed with obvious selectivity. This phenomenon demonstrates that the different development stages have different gene expression signatures. At the TD2 stage, some metabolic enzyme genes of auxin are upregulated, such as the *dioxygenase for auxin oxidation* gene (*DAO*), which encodes 2-oxoglutarate-dependent-Fe (II) dioxygenase, and the *IAA-amino acid hydrolase ILR1-like 6* gene (*ILL6*). The other DEGs are associated with auxin signal transduction, such as *ARF18*, *GH3.11*, *SAUR32*, and so on (Figure 6A). At the TG2 stage, more auxin-regulated DEGs are involved (metabolic: *YUCCA2* and *ILL7*; transport: *PIN3*, *PIN1*, *LAX2*, and *LAX3*; signal transduction: *ARF8*, *IAA27*, *AUX22E*, etc.) than in TD2 (Figure 6A). At this stage, auxin synthesis and the polar transport capacity are enhanced. In contrast, the genes of cytokinin biosynthesis, such as *IPT5* and *CYP735A2*, are highly expressed at the TD2 stage. The enzyme gene of the cytokinin transporter *PURINE PERMEASE 11* (*PUP11*) is also upregulated at this stage. At TG2, there are more cytokinin signaling genes than at TD2, such as *ARR15*, *ARR12*, *ARR9*, *ARR9*, *ARR8*, *ARR7*, *ARR3*, *AHK2*, and *AHK4*. *CKX5*, which catalyzes the oxidation of cytokinins, is also upregulated (Figure 6B).

Many transcription factors (TFs) from the TCP and WRKY families were annotated in the DEG profile (Figure 6C). From the D2 to TG2 stage, the expression pattern of most genes in the WRKY family showed a trend of “high–higher–low”, and most genes of the TCP family were “low–low–high”. In the DEG file, the number of genes from the MYB family was relatively small. The *MYB13* expression pattern was consistent with most genes in the WRKY family, and *MYB86* and *MYB39* were more like the TCP family genes. These results speculate that WRKYs may keep the bud dormant, and TCPs may facilitate early release. However, *WRKY71* is only highly expressed at TD2, and *BRC1* is only highly expressed at D2 (Figure 6C).

### 2.7. qRT-PCR Validation of Differentially Expressed Transcripts from Transcriptome Analysis

**Figure 7:**
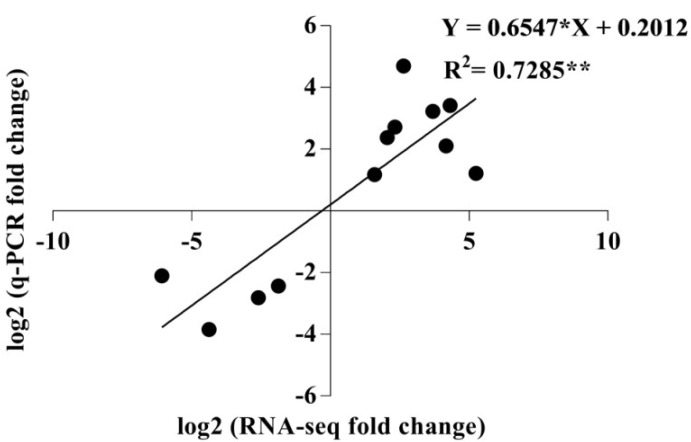
Comparison of expression levels measured by RNA-Seq and qRT-PCR for 12 selected differentially expressed genes for TG2 vs. D2 (nine DEGs) and TD2 vs. D2 (three DEGs).

To validate the accuracy of the transcriptome analysis, 12 DEGs were randomly selected for real-time RT-PCR analysis. The correlation between the RNA-Seq and qRT-PCR results was analyzed using GraphPad Prism 7.0 software. There was highly significant correlation (*r* = 0.8535) between the qRT-PCR and RNA-Seq-generated data (*p* < 0.01) (Figure 7), indicating that the transcriptome analysis was precise.

### 2.8. qRT-PCR Expression Analyses of Candidate Genes

**Figure 8:**
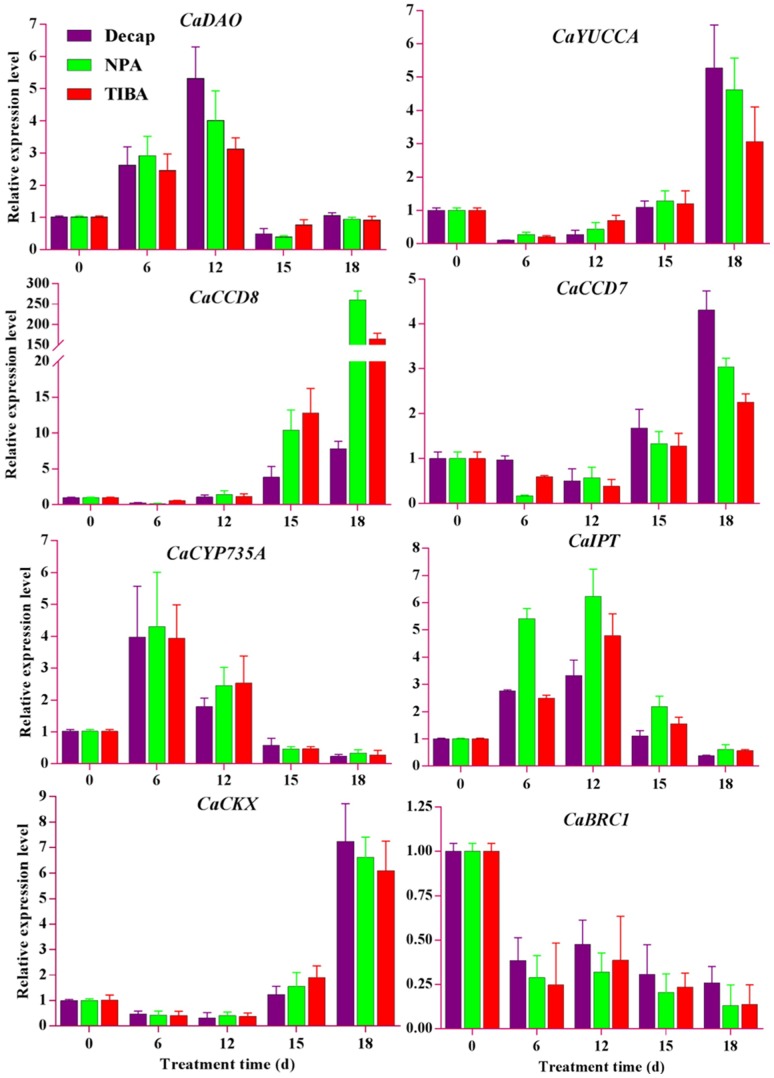

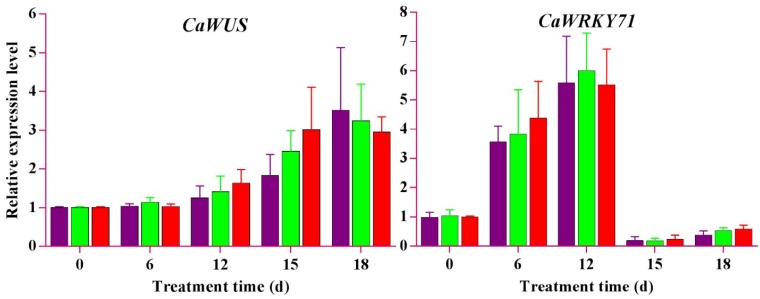
The relative expression levels of 10 candidate DEGs were measured during the lateral bud elongation process in *C. appendiculata* by qRT-PCR. Auxin synthesis and metabolic genes: *CaYUCCA* and *CaDAO*; cytokinin synthesis and metabolic genes: *CaCYP735A*, *CaIPT*, and *CaCKX*; strigolactone synthesis genes: *CaCCD7* and *CaCCD8*; and transcription factors: *CaBRC1*, *CaWUS*, and *CaWRKY71*. Values are means ± SD, *n* = 3. Error bars indicate the standard deviations obtained from three biological replicates.

To elucidate the relationship between hormone level changes and lateral bud elongation at the molecular level, we used auxin transport inhibitors (NPA and TIBA) and decapitation treatment to investigate the regulation mechanism of endogenous hormones on lateral bud break. Ten DEGs (seven plant hormone metabolism-related genes and three related transcription factors) were for the qRT-PCR analysis. The qRT-PCR results showed that decapitation, NPA, and TIBA treatments upregulated the expression of *CaDAO* at the transition stage (Figure 8), indicating that the auxin level decreased through the oxidative degradation pathway in the lateral buds. At this stage, the zeatin content rapidly increased due to the upregulation of *CaCYP735A* and *CaIPT* (Figure 8). On the sixth day of treatments, *CaCCD7* and *CaCCD8*, which are key genes for the synthesis of strigolactone, were inhibited (Figure 8). This might be related to the decreased levels of auxin. At the bud break stage, *CaCYP735A* and *CaIPT* were downregulated. In contrast, *CaYUCCA*, *CaCCD7*, *CaCCD8*, and *CaCKX* were upregulated (Figure 8). This is very likely to be related to the competition between growing buds. The synthesis of strigolactone in the growing shoots acts on the subordinate bud to consolidate its apical dominance, which is mediated by auxin.

It is well known that cytokinin regulates the expression of *BRC1* [41]. It has been previously stated that zeatin is at a high level during the bud break processes. Our results showed that expression of *CaWUS* (as a positive regulator to maintain meristematic cell activity) gradually increased (Figure 8), and *CaBRC1* (as a key negative regulator in shoot branching) was downregulated during the bud break processes (Figure 8). Obviously, a high cytokinin level promotes the expression of *CaWUS* and inhibits *CaBRC1* expression.

The DEG profiles showed that *CaWRKY71* was highly expressed at the TD2 stage, with little expression at the TG2 stage (Figure 7). The qRT-PCR results also showed that *CaWRKY71* was upregulated at the transition stage (Figure 8), and downregulated after treatment for 15 days. Interestingly, opposite trends were observed for the expression level of *CaWRKY71* and the IAA content (Figure 3C). This indicates that *CaWRKY71* might also be regulated by auxin.

## 3. Discussion

Shoot branching is a complex regulatory process. The transcriptome provides a good platform for revealing biological phenomena at the molecular level. We carried out a comprehensive transcriptome study at four critical developmental stages to reveal the molecular regulatory network. We particularly focused on the roles of auxin, cytokinin, and strigolactone in metabolism and signal transduction, as well as their interactions.

Shoot branching is regulated by both external and internal factors, such as light and phytohormones [42]. Thus far, two major hypotheses for the mechanism of auxin action in shoot branching have been developed: the second messenger theory and auxin transport/canalization. The auxin transport/canalization hypothesis states that lateral bud development is inhibited due to the inhibition of auxin transport from the lateral buds to the main stem [26,43,44,45]. In the second messenger theory, the auxin signal is relayed by several downstream messengers, such as CTKs [20] and SLs [46,47,48]. In our study, opposite trends were observed for the IAA content and the expression level of *CaDAO* (Figure 3A and Figure 8). It has been reported in the literature that NPA and TIBA have good inhibitory effects on auxin polar transport [49,50,51]. Thus, the reduction of auxin content is more likely achieved through metabolic pathways. At the transition stage, the cytokinin content was detected to gradually increase (Figure 3B), and, subsequently, the lateral buds sprouted (Figure 2). Our results also support the second messenger theory. In other words, auxin regulates lateral bud outgrowth by mediating cytokinin in this plant. 

Auxin was the first hormone found to regulate the growth of plant lateral buds [52]. It inhibits cytokinin synthesis by mediating the *AUXIN RESISTANT1* (*AXR1*) gene through its signal transduction pathway [20]. Studies have shown that auxin inhibits the expression of isopentenyl transferase (*IPT*)—a key gene of cytokinin synthetase—to control the synthesis of cytokinin, thereby inhibiting the growth of lateral buds [19]. Our results showed that the key enzyme gene for cytokinin synthesis was inhibited when the level of auxin was high at the D2 stage (Figure 3, Figure 6 and Figure 8). After decapitation, NPA, and TIBA treatments, the auxin level rapidly decreased (Figure 3A) and the expression of these genes was upregulated (Figure 8). At this time, the cytokinin content sharply increased (Figure 3B). Finally, the lateral buds grew into seedlings (Figure 2D–G). At the bud break stage, *CaYUCCA*—a key enzyme gene of auxin synthesis—was upregulated, resulting in increasing auxin levels (Figure 2A and Figure 7). However, *CaIPT* was downregulated, and the cytokinin level also decreased at this stage (Figure 3B). Our results confirm that auxin tends to control cytokinin biosynthesis to regulate the lateral bud elongation in this plant. At TG2, the auxin levels were enhanced, which might be related to the growth of lateral buds [15,26,43,44].

Cytokinin (CTK) is the primary hormone that positively regulates axillary bud outgrowth [18]. In *Arabidopsis thaliana*, *WUS* controls meristem function through direct regulation of cytokinin-inducible response regulators [53,54]. Experiments have illustrated that cytokinin upregulates the expression of *WUS* through *AHK2*- and *AHK4*-dependent pathways [55]. Dai et al. [56] proved that cytokinin-induced upregulation of *WUS* expression is due to *ARR12* binding to the promoter of *WUS*. Xie et al. [57] also confirmed that *WUS* expression is a cytokinin-dependent B-*ARR* target gene (*ARR12*) in shoot development. Obviously, cytokinin signal transduction is necessary for the activation of *WUS* expression. At the TD2 stage, cytokinin synthesis was significantly enhanced via the upregulation of *CaIPT* and *CaCYP735A* (Figure 7). Our results also showed that *CaWUS* was upregulated at the TD2 stage (Figure 7). As we expected, *AHK2*, *AHK4*, and *ARR12* were also highly expressed at TD2 (Figure 6B). Thus, the upregulation of *CaWUS* is also achieved by cytokinin-mediated signal transduction. It is very important that the *WUS* gene controls the number of stem cells [58,59]. During the process of organ formation, stem cells are continuously transformed into daughter cells, and then, these cells differentiate and form organs [60]. Thus, sustained high expression of *CaWUS* is required to balance the lost stem cells. We speculate that cytokinin promotes the expression of *CaWUS* to indirectly promote lateral bud outgrowth in this plant.

Cytokinin signaling is based on a two-component system that is achieved by the continuous transfer of phosphate groups between major components [61]. Auxin can directly activate the expression of *AAR7* and *ARR15*, and synergistic auxin antagonizes the action of cytokinin [62]. Chickarmane et al. [54] reported that *WUS* can regulate cytokinin synthesis through negative feedback. At the TG2 stage, A-*ARRs* transcription factors (*ARR3*, *ARR5*, *ARR7*, *ARR8*, *ARR9*, and *ARR15*) are highly expressed (Figure 6B), and the cytokinin level decreases. The cytokinin content decrease may be associated with the status of lateral bud growth. This also reflects the interactions between plant hormones to precisely regulate plant development.

Strigolactone acts as a downstream signal molecule of auxin. Auxin directly regulates the strigolactone level by controlling the expression of key synthase genes of strigolactone (two carotenoid cleavage dioxygenases (*CCD7* and *CCD8*)) [21,22,23,24,63]. *CCD7* and *CCD8* convert, together with the β-carotene isomerase D27, all-*trans*-*β*-carotene into carlactone, a key intermediate in the SL biosynthesis pathway [64,65]. In this study, *CaCCD7* and *CaCCD8* were downregulated when the ZT/IAA ratio gradually increased at the transition stage. Interestingly, the expression levels of these genes were upregulated when this ratio decreased (Figure 3A,C and Figure 8), indicating that strigolactone synthesis is associated with the ZT/IAA ratio. Strigolactone directly upregulates *BRC1* expression to inhibit lateral bud outgrowth [41]. Our data show that *CaBRC1* is highly expressed at the D2 stage (Figure 6C). Thus, the interaction of auxin, cytokinin, and strigolactone jointly regulates the the lateral bud break of *C. appendiculata*.

Transcription factors act as signal transduction switches to directly regulate plant growth and development [66]. There are many reports on *BRC1*’s regulation of branch development [67,68,69,70]. Our results also showed that BCR1 expression is downregulated, while zeatin remains at a relatively high level (Figure 3B and Figure 8). It has also been reported that *BRC1* inhibits lateral bud outgrowth through repressing cell cycle progression [71]. In this plant, cytokinins may have the same regulatory effects on *BRC1*. It is worth noting that high *BRC1* transcript levels do not guarantee that buds will not grow out [72]. However, further experimental demonstration is required to determine whether there is a negative feedback regulation mechanism for *BRC1*.

Some positive regulatory factors have been discovered in plant branch development, such as *WRKY71* [33] and *R2R3-MYB* [31]. In *Arabidopsis*, *WRKY71* positively regulates the transcription of *RAX1*, *RAX2*, and *RAX3* to initiate bud outgrowth [32]. Researchers believe that *WRKY71* regulates auxin homeostasis to control shoot branching. However, our results showed that *CaWRKY71* is inhibited at high auxin levels, whereas *CaWRKY71* expression is upregulated when auxin levels decrease (Figure 3A and Figure 8). This is inconsistent with previous reports from the literature.

## 4. Methods and Materials

### 4.1. Plant Materials, Growth Conditions, and Treatments

Unless stated otherwise, the experimental plants were triennial (with three pseudobulbs in the plant) and robust *Cremastra appendiculata* (D. Don) Makino which were grown in a glasshouse with temperature (15–22 °C) and humidity (70–85%) control. The cultivation medium was loose humus soil.

To investigate the molecular mechanism of the lateral bud break in *C. appendiculata*, its pseudobulb strings were treated to induce lateral bud break by decapitation (cutting off the annual pseudobulb), as previously described by Lv et al. [10], and with NPA and TIBA, as previously described by Ferguson and Beveridge [42] (Figure S7). The apical breaking buds were collected to be used as a transcriptome sample (G1 of Figure 2). Lateral buds collected at 0 (D2 of Figure 2), 6 (TD2 of Figure 2), and 18 days (TG2 of Figure 2) post-decapitation were taken as the other three transcriptome samples and used for qRT-PCR confirmation. The lateral bud samples from biennial pseudobulbs were collected after treatment for 0, 6, 12, 15, and 18 days to be used in the candidate gene expression analysis and hormone content analysis. Every treatment was executed in three biological replicates. All materials were snap-frozen in liquid nitrogen and stored at −80 °C until use.

### 4.2. RNA Isolation, Quantification, and Qualification

For bud samples, total RNA was isolated using the InnuPREP Plant RNA Kit (analytic-jena, Berlin, Germany). RNA degradation and contamination were monitored on 1% agarose gels. RNA purity was checked using the NanoPhotometer^®^ spectrophotometer (IMPLEN, Sacramento, CA, USA). RNA concentration was measured using Qubit^®^ RNA Assay Kit in the Qubit^®^ 2.0 Fluorometer (Life Technologies, Sacramento, CA, USA). RNA integrity (RIN ≥ 6.5) was assessed using the RNA Nano 6000 Assay Kit of the Agilent Bioanalyzer 2100 system (Agilent Technologies, Sacramento, CA, USA).

### 4.3. Library Construction and Sequencing

A total amount of 1.5 µg RNA per sample was used as input material for the RNA sample preparations. Sequencing libraries were generated using NEBNext^®^ Ultra™ RNA Library Prep Kit for Illumina^®^ (NEB, Lisbon, NH, USA) following the manufacturer’s recommendations, and index codes were added to attribute sequences in each sample. Briefly, mRNA was purified from total RNA using poly-Toligo-attached magnetic beads. Fragmentation was carried out using divalent cations under an elevated temperature in NEBNext First Strand Synthesis Reaction Buffer (5×). First-strand cDNA was synthesized using random hexamer primers and M-MuLV Reverse Transcriptase (RNase H). Second-strand cDNA synthesis was subsequently performed using DNA Polymerase I and RNase H. The remaining overhangs were converted into blunt ends via exonuclease/polymerase activities. After adenylation of the 3′ ends of the DNA fragments, a NEBNext Adaptor with a hairpin loop structure was ligated to prepare for hybridization. To preferentially select cDNA fragments of 150–200 bp in length, the library fragments were purified with the AMPure XP system (Beckman Coulter, Beverly, MA, USA). Then, 3 µL USER Enzyme (NEB, USA) was used with size-selected, adaptor-ligated cDNA at 37 °C for 15 min, followed by 5 min at 95 °C before PCR. Then, PCR was performed with Phusion High-Fidelity DNA polymerase, Universal PCR primers, and Index (X) Primer. Finally, PCR products were purified (AMPure XP system) and the library quality was assessed on the Agilent Bioanalyzer 2100 system. The library preparations were sequenced on an Illumina Hiseq. 2500 platform, and 150 bp paired-end reads (PE150) were generated by following the manufacturer’s recommendations.

### 4.4. De Novo Assembly and Annotation

The clustering of the index-coded samples was performed on a cBot Cluster Generation System using TruSeq PE Cluster Kit v3-cBot-HS (Illumia) in accordance with the manufacturer’s instructions. After cluster generation, the library preparations were sequenced on an Illumina Hiseq platform, and paired-end reads were generated. Raw data (raw reads) in fastq format were firstly processed through in-house perl scripts. In this step, clean reads were obtained using Trimmomatic (ver 0.30) [73] to remove the adapter, reads containing ploy-N, and low-quality reads from the raw reads. At the same time, the Q20, GC content, and the sequence duplication level of the clean data were calculated. High-quality clean reads were pooled and assembled with the short read de novo assembly program Trinity [74], with all parameters set to default values and minimum kmer_cov set to 2 by default. Trinity combined all clean reads with certain lengths of overlap to form longer fragments, called contigs, which were further linked to generate sequences that could not be extended further, known as unigenes. Additionally, the “completeness” of the assembly of these unigenes was assessed using BUSCO (ver 3.0.2) [75], and PCA analysis related diagrams were generated using ggplot2 packages in R (built-in function: prcomp).

To assign predicted gene descriptions, the assembled unigenes were aligned by BLASTx against the National Centre for Biotechnology Information (NCBI) non-redundant protein database (NR), nucleotide database (Nt), the Swiss-Prot protein database (Swiss-Prot), and the cluster of orthologous group database (COG), with a cutoff E-value < 1 × 10^−5^. ESTScan software was used to determine the sequence orientations for those unigenes which did not correspond to any of the above-mentioned databases. For functional annotation, the unigenes were examined against the Gene Ontology database (GO) using Blast2GO [76] (E-value < 1 × 10^−5^), The Pfam protein families database (Pfam) using HMMER 3.0 [77] (E-value < 1 × 10^−2^), and the Kyoto Encyclopedia of Genes and Genomes (KEGG) database using the KEGG automatic annotation server [78] (E-value < 1 × 10^−10^).

### 4.5. Identification of Differentially Expressed Genes

The Trinity-combined transcriptome was taken as a reference sequence (ref). Then, the clean reads of each sample were mapped to the ref. RSEM [79], with the bowtie2 parameter mismatch 0 (the bowtie2 default parameter); the results of the bowtie mapping were counted, and the number of read counts aligned to each gene for each sample was further obtained, followed by FPKM (expected number of Fragments Per Kilobase of transcript sequence per Millions base pairs sequenced) conversion to analyze the gene expression level [80]. Differential expression analysis of two groups was performed using the DESeq R package (1.10.1). DESeq2 [81] provides statistical routines to determine differential expression in digital gene expression data using a model based on the negative binomial distribution. The resulting *p*-values were adjusted using the Benjamini and Hochberg’s approach for controlling the false discovery rate. Genes with an adjusted *p*-value < 0.05 found by DESeq were identified as being differentially expressed. DEGs were then subjected to GO functional enrichment [82] and KEGG pathway analyses [78]. GO terms and KEGG pathways fulfilling the criterion of adjusted *p*-value < 0.05 were defined as being significantly enriched in DEGs. Gene expression data were normalized to 0, and DEGs were clustered by STEM [83]. Venn diagrams and heat maps were generated using Venn diagram and Pheatmap packages in R based on the gene list and the levels of gene expression for each bud type.

### 4.6. RNA-Seq Validation and Candidate Gene Expression Analysis Using qRT-PCR

For RNA-Seq validation, 1 µg of total RNA used in the previous RNA-Seq library construction was used for cDNA synthesis. For the candidate gene expression analysis, the cDNA was synthesized from 1 μg of total RNA for each sample. Each sample was collected from nine individual buds. A PrimeScript RT enzyme with a gDNA eraser (Takara, Japan) was used for cDNA synthesis. qRT-PCR was performed on an Applied CFX96 Real-Time PCR Detection System (Bio-Rad) using SYBR^®^Premix Ex Taq™ II (Takara, Tokyo, Japan). Primers designed from the conserved region of each cDNA were used for the qRT-PCR analyses (Tables S4 and S5). *C. appendiculata* actin (*CaActin*, Cluster-32503.44149) and elongation factor 1 *α* (*CaEf-1α*, Cluster-26967.95811) were used as the internal reference controls. The analysis was performed with three biological replicates. Relative expression levels (compared with 0 days) were calculated using the standard 2^−ΔΔ*C*t^ method. 

### 4.7. Measurements of Hormone Contents

Bud samples from five time points were used for hormone extraction and determination. Fresh buds (0.5 g) were used for IAA and ZT extractions, as described by Tarkowski et al. [84], with three biological replicates. Each sample was collected from 20 individual buds. The hormonal quantification was carried out using HPLC with a standard measure, as described by Ma et al. [85].

## 5. Conclusions

**Figure 9:**
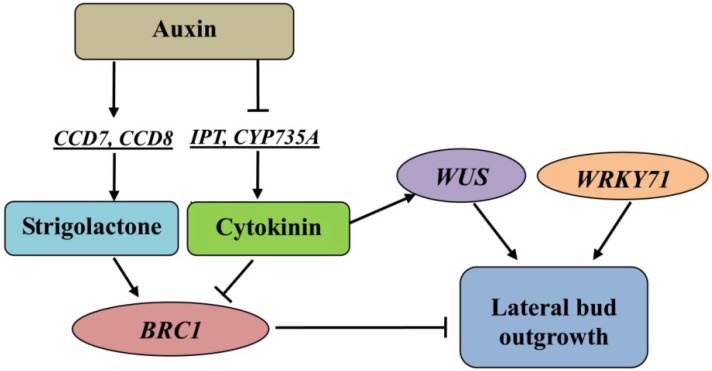
A hypothetical model to investigate the molecular mechanism of shoot branching in *C. appendiculata*. Auxin, cytokinin, and strigolactone might play crucial roles in regulating lateral bud outgrowth. *BRC1* acts as the integrator of these three hormones. Promotion and inhibition regulatory actions are indicated by arrows and lines with bars, respectively.

By combining the physiological and transcriptomic analyses, a hypothetical model was proposed to investigate shoot branching in *C. appendiculata* (Figure 9). Our formulated theories concerning the regulation of shoot branching and apical dominance adequately matched what was observed in annual plants. Auxin inhibits bud outgrowth and tends to inhibit cytokinin levels. Then, cytokinin promotes lateral bud burst by affecting the expression levels of the *WUS* and *BRC1* genes. The pseudobulb in the plant behaves in a same manner as that of a shoot above the ground. Therefore, this study lays the foundation for the molecular breeding of *C. appendiculata* and for studying the apical dominance of the underground stem.

## Figures and Tables

**Table 1:** Summary of sequences analysis.

Sample	Raw Reads	Clean Reads	Clean Bases (Gbp)	Q20 (%)	GC (%)	Mapped Rate (%)
**G1_1**	48,451,288	46,640,074	7.00	96.62	48.16	77.97
**G1_2**	53,695,306	51,981,244	7.80	97.19	49.33	79.33
**G1_3**	56,169,226	53,749,702	8.06	96.46	47.67	76.83
**D2_1**	56,176,712	53,803,124	8.07	97.54	47.01	74.20
**D2_2**	55,346,934	53,003,636	7.95	96.72	47.48	77.02
**D2_3**	50,324,224	48,136,950	7.22	97.54	46.71	71.16
**TD2_1**	46,568,478	45,272,406	6.79	97.39	47.49	72.62
**TD2_2**	48,746,182	46,913,750	7.04	96.18	46.33	69.36
**TD2_3**	45,843,728	44,538,914	6.68	96.91	46.57	71.04
**TG2_1**	49,637,968	48,035,784	7.21	97.71	49.57	73.10
**TG2_2**	54,961,460	53,625,420	8.04	96.58	48.75	74.09
**TG2_3**	52,872,172	51,352,168	7.7	96.25	49.1	74.15
**Summary**	618,793,678	597,053,172				
**Genes**	239,732					
**Mean length**	921 bp					
**N50 length**	1282 bp					

**Table 2:** Identification of over-represented GO term for phytohormone in DEG list.

GO Accession	GO Term	No. of Background Genes in This GO Term	NO. of Differentially Expressed Genes in This GO Term	Corrected *p* Value
	Biological process			
GO:0008207	C21-steroid hormone metabolic process	302	33	9.94 × 10^−3^
GO:0034754	cellular hormone metabolic process	359	35	2.18 × 10^−2^
GO:0042445	hormone metabolic process	406	36	4.12 × 10^−2^
GO:0010817	regulation of hormone levels	443	37	3.58 × 10^−2^
GO:0009755	hormone-mediated signaling pathway	190	15	3.53 × 10^−2^
GO:0003707	steroid hormone receptor activity	65	8	3.56 × 10^−2^
GO:0032870	cellular response to hormone stimulus	196	15	3.98 × 10^−2^
GO:0009725	response to hormone	486	29	4.79 × 10^−2^
GO:0016116	carotenoid metabolic process	282	33	6.20 × 10^−3^

## Supplementary Materials

Supplementary materials can be found online.
