# Supplementary material for: Transcriptome Profiles Reveal the Crucial Roles of Auxin and Cytokinin in the “Shoot Branching” of Cremastra appendiculata
Source: Int J Mol Sci. 2018 Oct 26;19(11):3354. doi: 10.3390/ijms19113354 (PMC6275059; doi:10.3390/ijms19113354)
Supplement: Supplementary file 1 [file ijms-19-03354-s001.pdf]

Supplementary Materials:

A. Figures

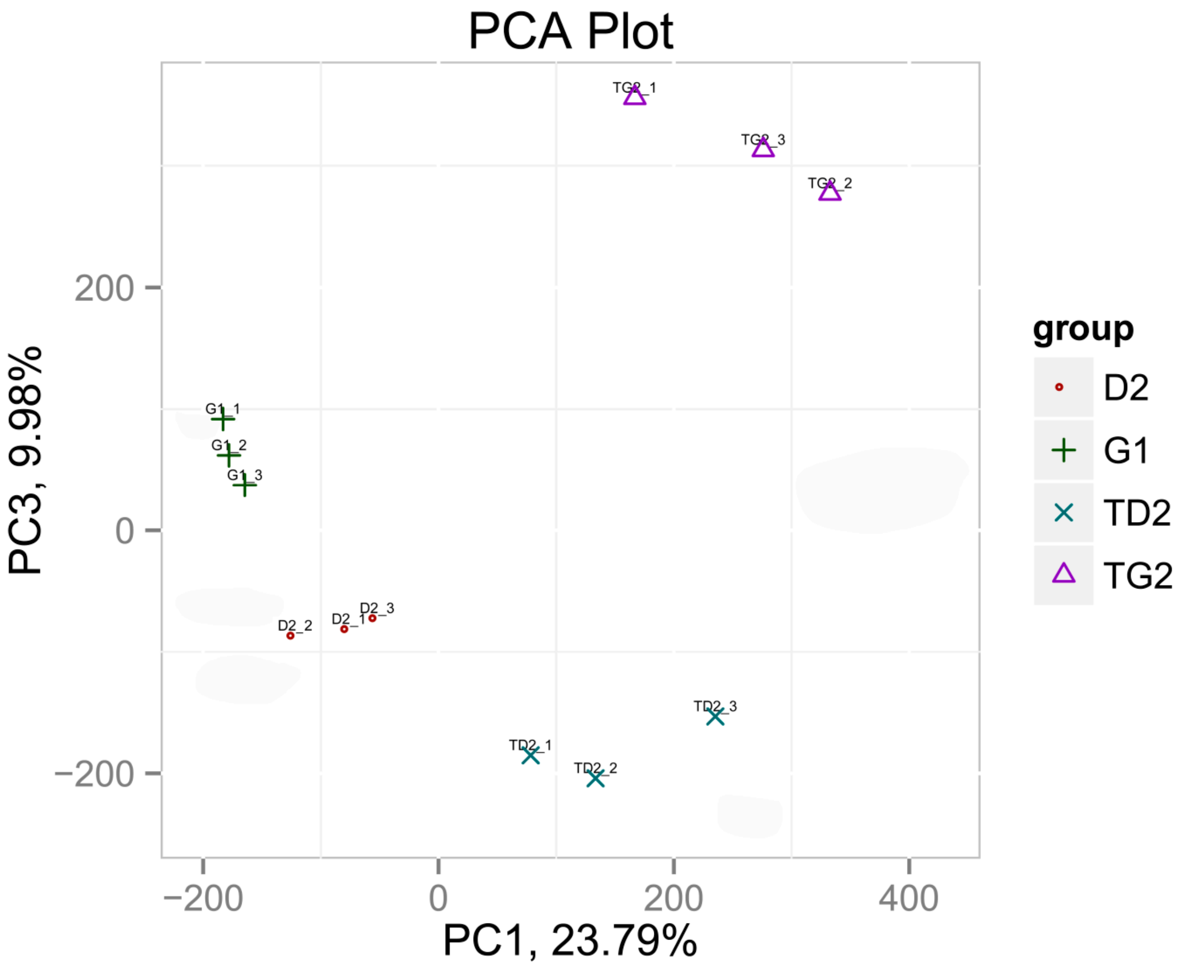

Figure S1 The PCA analysis separated 4 samples. The principal component 1 and the principal component 3 can explain difference of 23.79% and 9.98% for the four samples, respectively.

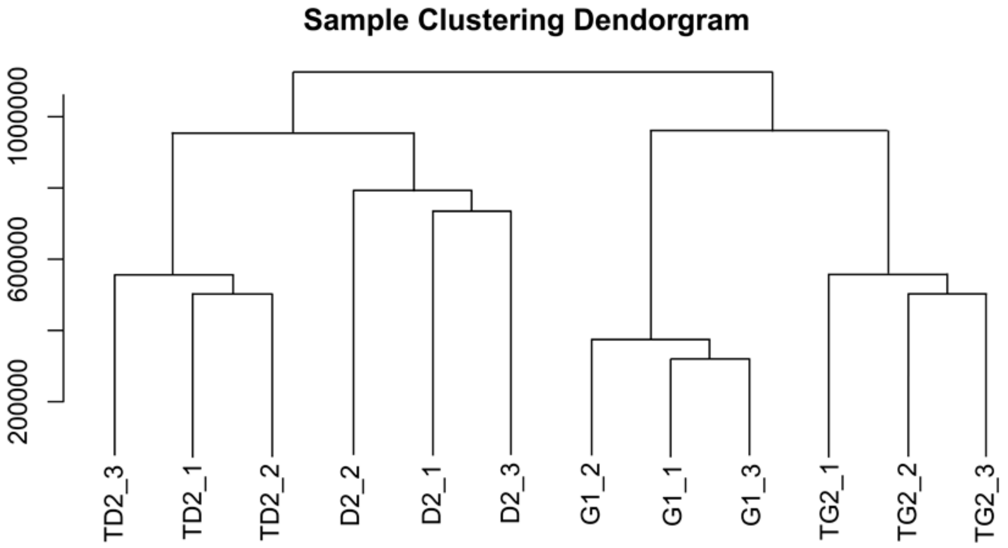

Figure S2 The PCA analysis obtained sample clustering dendorgram.

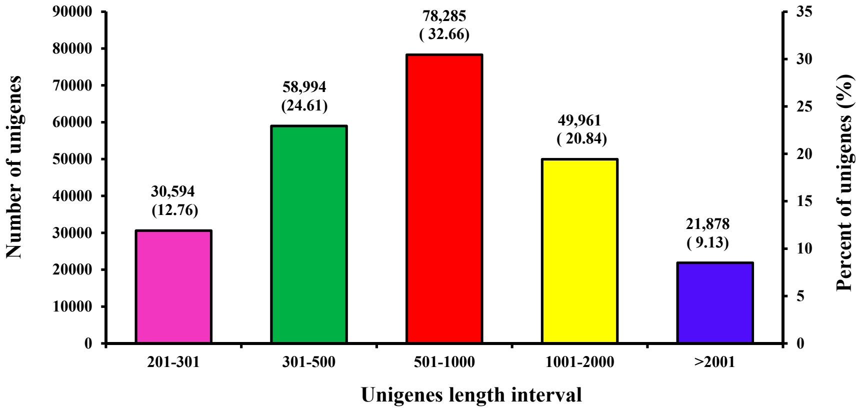

Figure S3 Length distribution of assembled unigenes.

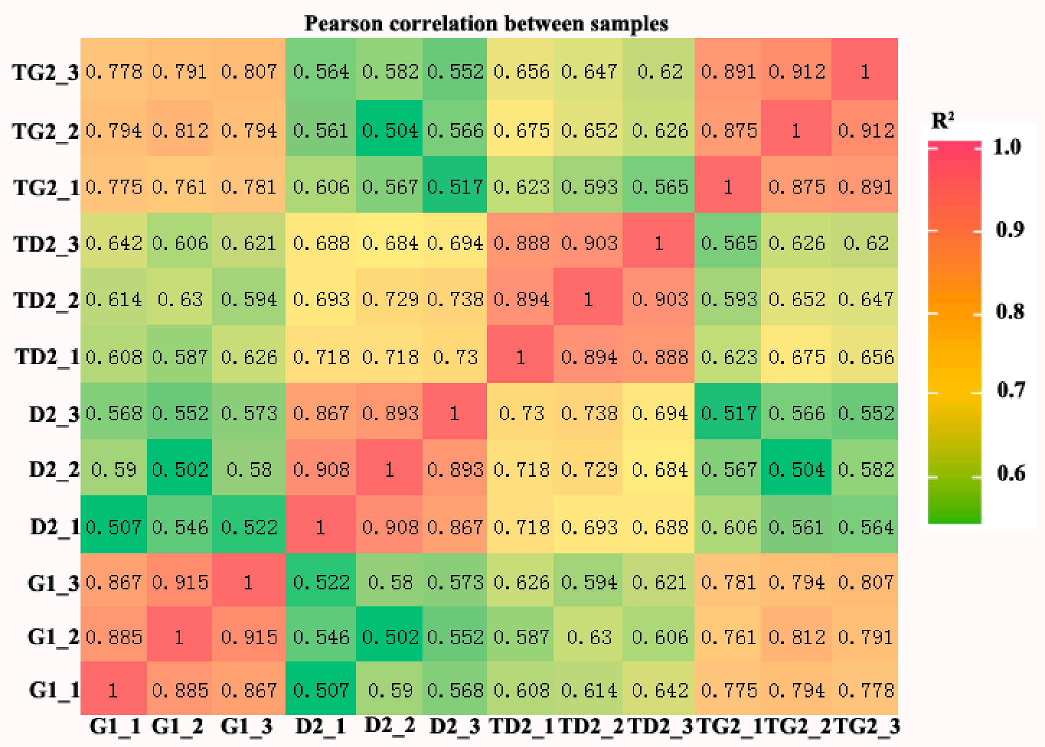

Figure S4 Correlation analysis of gene expression levels between samples. The numbers in the figure were the correlation coefficients obtained by the Pearson correlation calculation

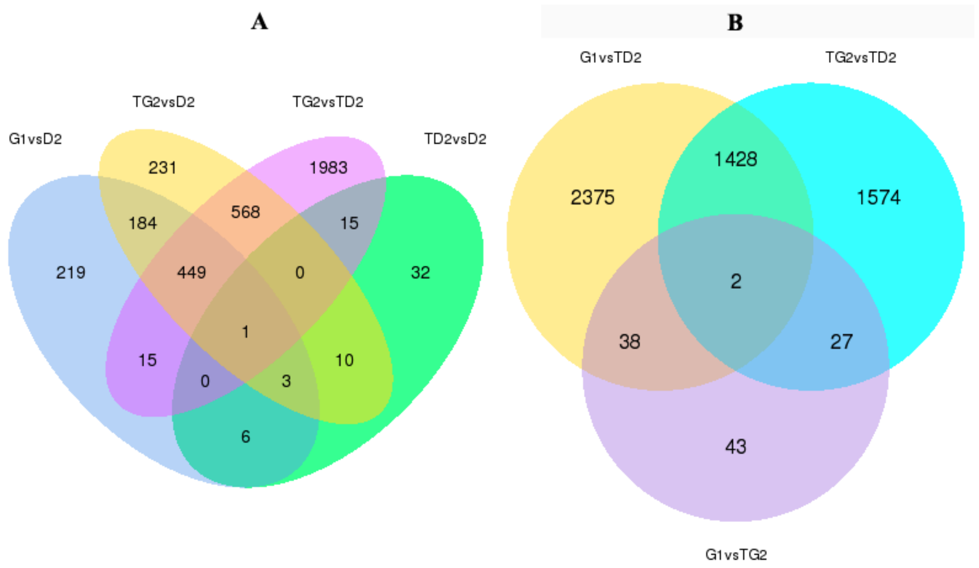

Figure S5 Venn diagram showing differentially expressed genes among groups. A: G1 vs D2, TG2 vs D2,TG2 vs TD2 and TD2 vs D2; B: G1 vs TD2, G1 vs TG2 and TG2 vs TD2.

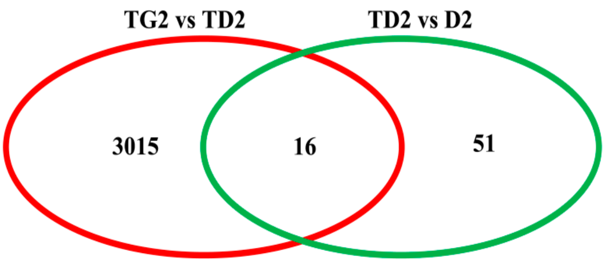

Figure S6 Venn diagram showing differentially expressed genes among TG2 vs TD2 and TD2 vs D2.

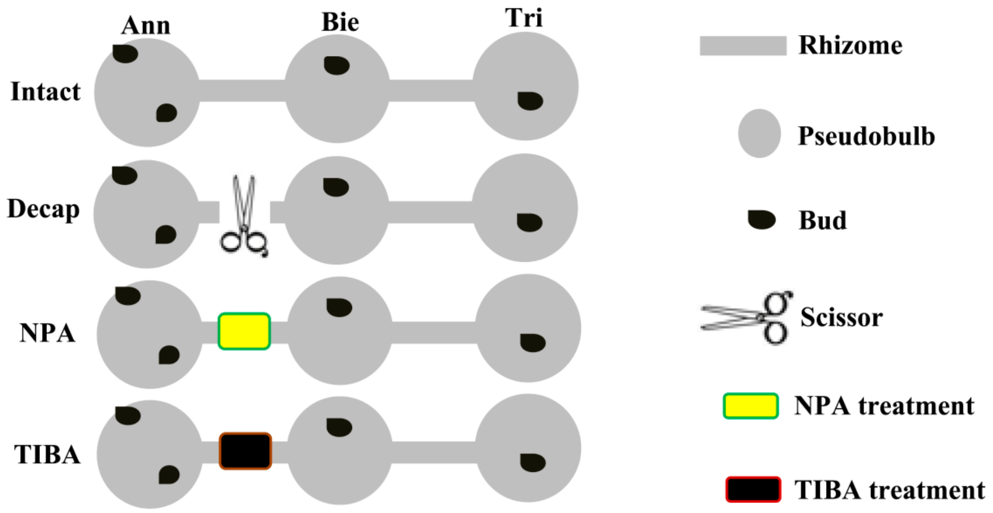

Figure S7 The samples treatments of mode pattern. **Ann**, **Bie** and **Tri** indicate annual, biennial and triennial pseudobulb of *C. appendiculata* respectively; **Intact**, **Decap**, **NPA** and **TIBA** indicate not treatment, Decapitation, N-1-naphthylphthalamic acid, and 2,3,5-triiodibenzoic acid respectively.

B. **T**ables

Table S1 Assessment of *C. appediculata* transcriptome assemblies using BUSCO

Number of genes	Evaluation items	ratio(%)
1247	Complete BUSCOs (C)	86.6
1157	Complete and single-copy BUSCOs (S)	80.3
90	Complete and duplicated BUSCOs (D)	6.3
58	Fragmented BUSCOs (F)	4.0
135	Missing BUSCOs (M)	9.4
1440	Total BUSCO groups searched	

Table S2 BLAST analysis of non-redundant unigenes against public databases.

Name	Number of Genes	Percentage (%)
Annotated in NR	110,579	46.12
Annotated in NT	55,635	23.20
Annotated in KO(KEGG)	41,991	17.51
Annotated in SwissProt	82,151	34.26
Annotated in PFAM	78,507	32.74
Annotated in GO	80,072	33.40
Annotated in KOG	32,164	13.41
Annotated in all Databases	15,199	6.33
Annotated in at least one Database	129,293	53.93
Total Genes	239,732	100

Table S3 DEGs summary.

	G1 vs D2	TD2 vs D2	TG2 vs D2	G1 vs TD2	G1 vs TG2	TG2 vs TD2
**Up**	665	44	1,148	1,998	65	1,984
**Down**	212	23	298	1,845	45	1,047
**Subtotal**	877	67	1,446	3,843	110	3,031
**Total**	5,988					

Table S4 primers sequences of selected twelve DEGs used in qRT-PCR validation.

Gene name	Gene ID	Sequences (5′–3′)	•	Product length/pb
CaActin	Cluster-32503.44149	F: TCCCAAGGCAAACAGAGAAAA<br>R: GGAAAGAACGGCTTGAATGG		91
CaEf-1α	Cluster-26967.95811	F: TGCCACACCTGCCACATTG<br>R: ATCCAGCATCTCCGTTCTTCAG		115
CaIPT	Cluster-26967.69023	F: CTTCGTCCTTGGCTCTTC<br>R: TGTTGGTGATGATGTGGAG		128
CaCCD7	Cluster-26967.62350	F: GCTGCTCTTCTCTTCCCAACC<br>R: CCATTTCCCTGTTTCCTCCTC		274
CaCCD8	Cluster-26967.101136	F: GGCTGGGTGCATTCGTTT<br>R: ACCCTCGTCATCTTCCTCCTC		249
CaTIR1	Cluster-32503.41867	F: ATGCTTTCGGTCGCCTTC<br>R: CCGCTCGTCCATAACCTCTAC		246
CaAUX1	Cluster-26967.30454	F: ATCCGAAGCTACCGCAAGAA<br>R: TTCAGTCAGCCCATCTCTACCA		252
CaAUX1/IAA	Cluster-26967.79918	F: CTCAGCGGCATCCTGTTTC<br>R: TTCTCCCTCTCCTTCCTTGTTCT		107
CaCRE1	Cluster-26967.95633	F: GAGGCTGCTCCGTTTGACTT<br>R: AATGCCCCTTCTTCTCCTTTTC		274
CaA-ARR	Cluster-26967.30111	F: GAAGATTAAGGGAGAGATGGGAGAG<br>R: AAGAGAATCATCCACCACCAAAA		103
CaTPS	Cluster-32503.46593	F: GCTATGAACGAAGCAATCTCCA<br>R: GCTACCACCCTGAAACCAAATC		194
CaUGDH	Cluster-32503.44357	F: CAAGAAAGACACGGGCGATAC<br>R: CTCTGAAAATCCAACTCCCTGAA		294
CaYUCCA	Cluster-26967.32402	F: CTGTATTGCCTCGCTATG<br>R: GAACACTGGACGAACTTC		181
CabglX	Cluster-32503.53197	F: CTGTCTCTCAGGCGGTCGT<br>R: GTGTAGCCAGAGCCCCATTC		151

Notes.*Ca* is abbreviation of *Cremastra appendiculata*; *CaActin* and *CaEf-1α* are housekeeping genes. The same below.

Table S5 Primers sequences of ten candidate DEGs used in qRT-PCR expression analysis.

Gene name	Gene ID	Sequences (5′–3′)	Product length/pb
CaActin	Cluster-32503.44149	F: TCCCAAGGCAAACAGAGAAAA<br>R: GGAAAGAACGGCTTGAATGG	91
CaEf-1α	Cluster-26967.95811	F: TGCCACACCTGCCACATTG<br>R: ATCCAGCATCTCCGTTCTTCAG	115
CaIPT	Cluster-26967.69023	F: CTTCGTCCTTGGCTCTTC<br>R: TGTTGGTGATGATGTGGAG	128
CaCCD7	Cluster-26967.62350	F: GCTGCTCTTCTCTTCCCAACC<br>R: CCATTTCCCTGTTTCCTCCTC	274
CaCCD8	Cluster-26967.101136	F: GGCTGGGTGCATTCGTTT<br>R: ACCCTCGTCATCTTCCTCCTC	249
CaBRC1	Cluster-26967.93695	F: GAGGAAGGATAGGCATAGC<br>R: CCATTGAACGGTCTTACTTG	142
CaYUCCA	Cluster-26967.32402	F: CTGTATTGCCTCGCTATG<br>R: GAACACTGGACGAACTTC	181
CaDAO	Cluster-26967.157097	F: GCTTGAACGATTGTTAGAGG<br>R:CTGGAATGAACGGCTCAC	199
CaCYP735A	Cluster-26967.63099	F:AGAGGAGAACCACTTAGC<br>R:ATGCCAACCACATCAATC	182
CaWUS	Cluster-26967.91579	F:GTCAGATTCAGGCAAGTC<br>R:CGAAGCACCATAATAAGAAC	185
CaCKX5	Cluster-26967.46933	F:TCCTCCTTCTTCTCCACTAG<br>R:CTCAACCTCCTCATCAATAG	144
CaWRKY71	Cluster-26967.116760	F:AAAGGAGAGAAGCGGCAAAG<br>R:GGTAACCGTCTTCTAGGTGATC	99
